# Mopping up crystals to keep the blood flowing

**DOI:** 10.1016/j.ebiom.2021.103786

**Published:** 2021-12-25

**Authors:** Christoph E. Hagemeyer

**Affiliations:** NanoBiotechnology Laboratory, Australian Centre for Blood Diseases, Central Clinical School, Monash University, Melbourne, Victoria 3004, Australia

Despite substantial progress in the prevention and treatment of cardiovascular diseases, mortality and morbidity remain unacceptably high; stroke and myocardial infarction are still one of the biggest killers worldwide.^1^ One hallmark of a ruptured atherosclerotic plaque causing a clinical event is the presence of cholesterol crystals (CC) that are fuelled by high plasma cholesterol levels. These deposits are extremely pro-inflammatory which contributes to plaque inflammation, instability and rupture.

Primary preventions and treatments have focused on lipid-lowering drugs and, more recently, anti-inflammatory drugs.^2^ Despite encouraging results, some patients still suffer fatal events due to CC triggered inflammation that is already well established before treatment is initiated. One avenue to address this shortcoming is to remove the culprit altogether even after it has taken hold of the vessel wall. However, no effective treatment is currently available to dissolve and remove CC from plaques to eliminate this major cause of plaque inflammation.

Nanomedicine, a rapidly developing field with some remarkable preclinical achievements so far, holds a lot of promise in the delivery of more potent and specific therapies for atherosclerosis.^3^ The range of activities in the field has significantly increased over the last decade thanks to substantive investments and the emergence of new companies focusing exclusively on nanotechnology. In recent years, there has been a clear trend to use particle systems inspired by nature. Given its prominent role in lipid metabolism, high-density lipoprotein (HDL) is such a promising system that has been widely used in reconstituted form or as a design template for engineered particles. Unfortunately, clinical success and translation have been lacking so far despite some promising preclinical success stories.^4^ The most advanced clinical product is CSL112 which has interestingly been proposed as an acute anti-inflammatory drug after myocardial infarction^5^ and not for the atherosclerotic plaque treatment indication.

In a recent issue of EBioMedicine, Luo and colleagues present a novel engineered HDL-like phospholipid-based nanoparticle, termed ‘Michigan Nanoparticle’ (miNano).^6^ The new nanomedicine shows a remarkable ability to bind to and dissolve CCs as well as remove excess cholesterol from the plaque. Consequently, inflammation in the vessel wall was reduced and atherosclerosis was inhibited when compared to reconstituted HDL. This was directly attributed to the prevention of foam cell formation and reduced macrophage infiltration. The team also made some preliminary attempts toward translation by demonstrating CC removal from human plaques ex vivo. Interestingly, even without actively targeting the miNano particles accumulated in the diseased vessel wall and not in healthy vessels. This is a clear advantage for clinical translation compared to other systems as it reduces the complexity and cost of goods.

There are a few limitations associated with the study. The team used Polyethylene Glycol (PEG) to ensure the engineered particles are sufficiently low fouling and are not recognised by the immune system. Although FDA approved, PEG is non-biodegradable and can accumulate in tissue with unknown long-term consequences. In addition, PEG antibodies are highly prevalent in the general population.^7^ This is potentially problematic for a therapy that very likely needs to be given lifelong to achieve control of cardiovascular disease. Amino-acid based PEG alternatives could be the way forward.^8^ The second limitation is the animal model used. While preclinical models of atherosclerosis develop plaques, most don't show a vulnerable phenotype as seen in human disease. Next-generation models such as the Tandem Stenosis model that develop uniquely “human-like” plaques with hallmarks of vulnerability such as vasa vasorum, intraplaque haemorrhage and ruptured fibrous caps^9^ could be employed before moving towards clinical translation.

Cholesterol accumulation and the formation of CC is not only seen in atherosclerotic plaques but also several other conditions including non-alcoholic steatohepatitis (NASH), another epidemic health burden.^10^ It is interesting to speculate how the miNano particles could help remove cholesterol from the liver and reduce the inflammatory burden on this vital organ. In contrast to the plaque application, active targeting of the liver or the removal of the added low fouling elements (which are designed to direct nanoparticles away from the liver) in the current version of miNano is likely required to achieve specific liver accumulation. It is also intriguing to postulate loading miNano with other molecules (anti-inflammatory drugs, therapeutic oligonucleotides, etc.) to further enhance the potency of the drug delivery system.

Overall, the study highlights the importance of developing novel therapies for cardiovascular disease that could potentially further reduce this large health burden. As with all preclinical studies, further work is required moving from additional preclinical efficacy testing in large animals to long term safety studies. The 6-week safety testing results presented are encouraging, but for something that needs to be administered lifelong, more studies are likely required.

## Declaration of interests

The author declares no conflict of interest.

## Appendix Supplementary materials

Image, application 1
